# *Galleria mellonella* Model Identifies Highly Virulent Strains among All Major Molecular Types of *Cryptococcus gattii*

**DOI:** 10.1371/journal.pone.0105076

**Published:** 2014-08-18

**Authors:** Carolina Firacative, Shuyao Duan, Wieland Meyer

**Affiliations:** 1 Molecular Mycology Research Laboratory, Centre for Infectious Diseases and Microbiology, Sydney Medical School – Westmead Hospital, Marie Bashir Institute for Infectious Diseases and Biosecurity, The University of Sydney, Westmead Millennium Institute, Sydney, Australia; 2 Grupo de Microbiología, Instituto Nacional de Salud, Bogotá, Colombia; University of Minnesota, United States of America

## Abstract

Cryptococcosis is mainly caused by *Cryptococcus neoformans*. However, the number of cases due to *C. gattii* is increasing, affecting mainly immunocompetent hosts. *C. gattii* is divided into four major molecular types, VGI to VGIV, which differ in their host range, epidemiology, antifungal susceptibility and geographic distribution. Besides studies on the Vancouver Island outbreak strains, which showed that the subtype VGIIa is highly virulent compared to the subtype VGIIb, little is known about the virulence of the other major molecular types. To elucidate the virulence potential of the major molecular types of *C. gattii*, *Galleria mellonella* larvae were inoculated with ten globally selected strains per molecular type. Survival rates were recorded and known virulence factors were studied. One VGII, one VGIII and one VGIV strain were more virulent (*p* <0.05) than the highly virulent Vancouver Island outbreak strain VGIIa (CDCR265), 11 (four VGI, two VGII, four VGIII and one VGIV) had similar virulence (*p* >0.05), 21 (five VGI, five VGII, four VGIII and seven VGIV) were less virulent (*p* <0.05) while one strain of each molecular type were avirulent. Cell and capsule size of all strains increased markedly during larvae infection (*p* <0.001). No differences in growth rate at 37°C were observed. Melanin synthesis was directly related with the level of virulence: more virulent strains produced more melanin than less virulent strains (*p* <0.05). The results indicate that all *C. gattii* major molecular types exhibit a range of virulence, with some strains having the potential to be more virulent. The study highlights the necessity to further investigate the genetic background of more and less virulent strains in order to recognize critical features, other than the known virulence factors (capsule, melanin and growth at mammalian body temperature), that maybe crucial for the development and progression of cryptococcosis.

## Introduction

The sibling species *Cryptococcus neoformans* and *C. gattii* are the cause of cryptococcosis, a life-threatening invasive infection that compromises the respiratory and/or central nervous systems. When caused by *C. neoformans*, cryptococcosis appears as an opportunistic infection mainly affecting HIV-positive patients, whereas cryptococcosis caused by *C. gattii* occurs more frequently in immunocompetent hosts [1]–[4]. Four major molecular types of each species have been identified using different molecular methods, VNI to VNIV for *C. neoformans* and VGI to VGIV for *C. gattii* [5]–[9]. Although the majority of the cases of cryptococcosis worldwide are caused by *C. neoformans* molecular type VNI, *C. gattii* has emerged as a primary pathogen in recent years, by expanding its geographic distribution and environmental niche, and causing fatal infections in humans, domestic and wild animals. The most prominent outbreak is the ongoing outbreak on Vancouver Island, British Columbia, Canada, due to isolates of two subtypes of the *C. gattii* molecular type VGII [10]. Based on virulence studies in a murine model, the outbreak strains of the major subtype VGIIa were recognized to be significantly more virulent than the strains of the minor subtype VGIIb [11]. Since then, several outbreaks and isolated but fatal cases caused by the molecular types VGI [12], [13], VGII [14], [15] and VGIII [16], [17] but not VGIV have been described. This is most likely due to the fact that VGIV has rarely been isolated. To date, only few clinical and environmental isolates of *C. gattii* molecular type VGIV have been reported, all from Central and South America, Asia and South Africa [2], [7], [18]–[23].

Besides the differences in geographical distribution and epidemiology of the major molecular types of *C. gattii*, some studies indicate that there is an association between the molecular type and susceptibility to different antifungal drugs, with VGII strains being less susceptible compared with strains of the other *C. gattii* molecular types and *C. neoformans* [24]–[26]. In addition, using a *Drosophila* model, molecular type-specific differences in *C. gattii* (grown at 30°C) were found recently, with VGIII being the most virulent molecular type [27]. However, the relationship between the molecular types of *C. gattii* and the virulence of strains at mammalian body temperature remains undetermined.

To study the virulence of the strains causing cryptococcosis, different *in vitro* and *in vivo* models have been employed. Models using mammals as the host are most commonly used, especially the model based on the intranasal inoculation of mice (*Mus musculus*), which most closely resembles naturally-occurring infection [28]. Non-mammalian models have been used to understand virulence *per se* and also other aspects of fungal pathogenesis, including individual virulence traits of the strains and the antifungal drug activity. The invertebrate model that uses the larvae of the wax worm *Galleria mellonella* (Lepidoptera) has been successfully employed in a number of virulence studies of clinically important fungi including *C. neoformans* [29]–[31], *C. gattii* [12], [32], the yeasts *Candida albicans* [33], [34] and *Candida krusei* [35] and the filamentous fungus *Aspergillus fumigatus* [36]. This model has shown a positive correlation when the pathogenicity of microorganisms in the larvae is compared with that in vertebrate mammalian models. In addition, the ease of inoculating the larvae with specific concentrations of the fungal pathogen, low maintenance costs of the larvae, the ability to keep the larvae under various temperature conditions, from 25°C to 37°C, and a lack of ethical limitations, reinforces its use.

Until now, this model of infection has only been used to study the virulence of four selected VGI strains in connection with the first reported case of *C. gattii* in South-eastern USA [12], and to test the effects on virulence after gene knockout studies [32]. A systematic study of all major molecular types of *C. gattii* is still missing. Therefore, the aim of the current study was to use *G. mellonella* larvae to determine the virulence of clinical, environmental and veterinary strains of all major molecular types of the emerging pathogen *C. gattii*, including those rarely recovered. The use of an invertebrate model permits pre-screening of more and less virulent strains for subsequent detailed molecular studies, aimed at identifying additional virulence factors involved in cryptococcal pathogenicity. Such factors could potentially be used for the diagnosis or therapy of cryptococcosis.

## Materials and Methods

### Strains and molecular typing

To evaluate the virulence of the major molecular types of *C. gattii*, 40 globally selected strains (10 per molecular type) were studied, including the well-characterised high (CDCR265) and low (CDCR272) virulent strains, representing the subtypes VGIIa and VGIIb form the Vancouver Island outbreak, respectively [11], [37]. From the strains, 27 were obtained from clinical, seven from environmental and six from veterinary sources. Among those, two clinical, two environmental and one veterinary strain were mating type a. Mating type and molecular type of the isolates was previously identified by conventional methods [6], [38]. Multilocus Sequence Typing (MLST) of the isolates had been carried out by using the ISHAM *C. neoformans* and *C. gattii* consensus MLST scheme, comprising seven genetic loci (*CAP59*, *GPD1*, *LAC1*, *SOD1*, *URA5*, *PLB1* and IGS1) [8]. Allele and sequence types were identified according to the mlst.mycologylab.org webpage. General information concerning the strains is shown in Table 1. All studied isolates are maintained at the Westmead Hospital Culture Collection of the Molecular Mycology Research Laboratory, Centre for Infectious Diseases and Microbiology, University of Sydney, Westmead Millennium Institute, Westmead, Australia.

**Table 1:** *Cryptococcus gattii* strains studied, including general information.

WM number	Other collection number	Country	Source/Mating type	Molecular type (ST)	Reference
WM 179	CBS 10078, IFM 50893 (VGI Standard)	Australia	Clin/alpha	VGI (ST51)	[6], [40]
WM 276	TCS-SC1	Australia	Env/alpha	VGI (ST154)	[50]
WM 352	IUM 96-2795	Italy	Env/alpha	VGI (ST147)	[40]
WM 834		Papua New Guinea	Clin/alpha	VGI (ST151)	[40]
WM 1243		Papua New Guinea	Clin/alpha	VGI (ST53)	[9]
WM 1899	IMIM 50A, LA 175, Spa-E2	Spain	Clin/alpha	VGI (ST103)	[6]
WM 2540	TP1414, Kiwi	New Zealand	Vet/alpha	VGI (ST52)	[50]
WM 2634	MCS022	India - Thailand	Clin/alpha	VGI (ST54)	[9]
WM 02.103	Cr 10	Argentina	Env/alpha	VGI (ST157)	[6], [40]
WM 05.410	LMM 244	Brazil	Clin/alpha	VGI (ST58)	[40]
WM 178	CBS 10082, IFM 50894 (VGII Standard)	Australia	Clin/alpha	VGII (ST21)	[6], [15], [40]
WM 02.32	CDC R265 (VGIIa Standard)	Canada	Clin/alpha	VGII (ST20)	[10], [11], [15]
WM 02.209	F 3179	Canada	Clin/alpha	VGII (ST20)	[10], [37]
WM 05.229	Bandiaga	Australia	Clin/alpha	VGII (ST7)	[15], [37], [50]
WM 05.342	H0058-I-1674	Colombia	Env/a	VGII (ST25)	[19], [37]
WM 06.10	LA 295, HM 143839, Arg-C10	Argentina	Clin/alpha	VGII (ST20)	[6]
WM 06.13	CBS 7750	USA	Env/alpha	VGII (ST20)	[10], [15]
WM 06.25	CDC R272 (VGIIb Standard)	Canada	Clin/alpha	VGII (ST7)	[10], [11], [15]
WM 06.634	DMST 20765	Thailand	Clin/alpha	VGII (ST7)	[37]
WM 06.636	DMST 20767	Thailand	Clin/alpha	VGII (ST7)	[37]
WM 175	CBS 10081 (VGIII Standard)	USA	Env/alpha	VGIII (ST60)	[6], [37]
WM 2088	H0058-I-1134, LA 622	Colombia	Clin/a	VGIII (ST59)	[6]
WM 2423	CN043	New Zealand	Clin/a	VGIII (ST68)	[49]
WM 02.139	NIH 198	USA	Clin/alpha	VGIII (ST93)	[10]
WM 09.47	08-7686	USA	Vet/alpha	VGIII (ST74)	Current study
WM 10.17	09-11987	USA	Vet/alpha	VGIII (ST76)	[40]
WM 11.8	H0058-I-2728	Colombia	Env/a	VGIII (ST116)	Current study
WM 11.105	H0058-I-2023	Colombia	Clin/alpha	VGIII (ST79)	Current study
WM 11.118	H0058-I-2961	Colombia	Clin/alpha	VGIII (ST146)	Current study
WM 11.139	JS110	USA	Vet/a	VGIII (ST143)	Current study
WM 779	CBS 10101 (VGIV Standard)	South Africa	Vet/alpha	VGIV (ST70)	[6], [9], [27]
WM 780	V00709	South Africa	Clin/alpha	VGIV (ST105)	[9]
WM 2363	B5742, M 30826	India	Clin/alpha	VGIV (ST69)	[9], [27]
WM 2570	M27046; P2244	South Africa	Clin/alpha	VGIV (ST158)	[40]
WM 2579	M27056; P2238	South Africa	Clin/alpha	VGIV (ST158)	[9]
WM 2604	M31499, 4357	South Africa	Clin/alpha	VGIV (ST160)	[40]
WM 2876	V00869	South Africa	Clin/alpha	VGIV (ST104)	[9]
WM 04.20	M27055, 25229	South Africa	Clin/alpha	VGIV (ST224)	[9]
WM 08.314	16-1664	Australia	Vet/alpha	VGIV (ST150)	[40]
WM 12.43	01-201083	Australia	Clin/alpha	VGIV (ST107)	Current study

Clin: clinical; Env: environmental; Vet: veterinary.

### Galleria mellonella model

*G. mellonella* larvae used in this study were obtained after the oviposition of the adult moths reared and maintained at 26°C and 60% relative humidity in the insectarium of Westmead Hospital Animal Care Facility, Sydney, Australia. Ten similar sized larvae (about 3 g each) were selected, placed in a 90 mm plastic Petri dish, weighed, and used for inoculation. Each *C. gattii* strain was previously grown on Sabouraud agar for 48 h at 27°C. Using a Neubauer Chamber, an inoculum of 10^8^ yeast cells/ml was prepared in Phosphate Buffered Saline (PBS) and 10 µl were inoculated into the hemocoel of each larva by injection into the last left pro-leg, using a 50U Insulin Syringe with a 29-gauge needle. A group of 10 larvae was also inoculated with PBS to monitor potential effects on survival due to physical injury, while another 10 were not inoculated at all as an untreated control. After injection, the larvae were incubated in Petri dishes at 37°C for 10 days and checked daily for any mortality.

### Cellular and capsular size of *C. gattii* after inoculation

Cells of all *C. gattii* strains were isolated from *G. mellonella* larvae after inoculation, to assess capsule production and cellular growth *in vivo*. Each dead larva was crashed and homogenized in 1 ml of PBS. Homogenates were filtered using nylon cell strainers (100 µm pore size; BD Falcon) and 10 µl of the cell suspension was stained with India ink (BD), observed using conventional microscopy and photographed. Cell and capsule sizes were measured directly under the microscope (Olympus, VANOX). Capsule size was estimated as the difference between the diameter of the total cell and the cellular body. Cell and capsule sizes of the strains were also measured immediately before inoculation, by India ink staining of the cell suspensions used for inoculation, prepared from *C. gattii* cells grown on Sabouraud agar for 48 h at 27°C.

### Melanin production of the strains

Laccase activity was quantified as previously described [39], with minor modifications. Each cryptococcal strain was inoculated in yeast nitrogen base medium (YNB) containing 1% glucose and 10 mM dopamine and grown for 48 h at 37°C and 250 rpm. Cultures were centrifuged, and the optical density (OD) of the supernatant was read using a spectrophotometer at 475 nm.

Melanin production was also assessed by growing each strain on Niger seed (*Guizotia abyssinica*) agar, for 48h at 37°C [39]. Melanin-producing strains make brown pigment on this medium.

### Growth test at 37°C

Yeast peptone-dextrose broth with 2% glucose was inoculated with 10^5^ *C. gattii* cells/ml (early-logarithmic phase), with initial OD at 600 nm of 0.01, and incubated at 37°C and 250 rpm. Cell density was determined by spectrophotometer using the OD_600_ after 18, 36 and 72 hours of incubation [37].

### Statistical analysis

Survival curves per strain were graphed, median survival times were calculated and estimation of differences in survival was analysed by the Log-rank (Mantel-Cox) test (recommended). When more than five larvae (50%) were alive at the end of the experiment, median survival times were not determined (ND) (Table 2).

**Table 2:** Values comparing survival curves and median survival times of *Galleria mellonella* larvae after being infected with the highly virulent strain of *Cryptococcus gattii* CDCR265 (VGIIa) and other strains of the molecular types VGI, VGII, VGIII and VGIV.

Strain	Molecular type	Source/Mating type	Number of deaths	Median survival time (h)	*p-*value	Virulence*
WM 05.229	VGIIb	Clin/alpha	10	48	< 0.0001	+++
WM 11.105	VGIII	Clin/alpha	10	84	0.0009	+++
WM 779	VGIV	Vet/alpha	10	96	0.0258	+++
WM 276	VGI	Env/alpha	10	108	0.1118	++
WM 780	VGIV	Clin/alpha	10	108	0.1488	++
WM 834	VGI	Clin/alpha	10	108	0.2589	++
WM 2088	VGIII	Clin/a	10	108	0.6061	++
WM 11.139	VGIII	Vet/a	10	108	0.2986	++
CDCR265#	VGIIa	Clin/alpha	10	120	NA	++
WM 06.10	VGIIa	Clin/alpha	10	120	0.7865	++
WM 06.636	VGIIb	Clin/alpha	10	120	0.4191	++
WM 09.47	VGIII	Vet/alpha	10	120	0.9269	++
WM 11.118	VGIII	Clin/alpha	10	120	0.639	++
WM 179	VGI	Clin/alpha	10	132	0.6565	++
WM 1243	VGI	Clin/alpha	10	132	0.2286	++
WM 352	VGI	Env/alpha	10	144	0.0289	+
WM 02.209	VGII	Clin/alpha	10	144	0.005	+
WM 2634	VGI	Clin/alpha	10	156	0.0031	+
WM 12.43	VGIV	Clin/alpha	10	168	0.0002	+
WM 1899	VGI	Clin/alpha	10	180	< 0.0001	+
WM 2579	VGIV	Clin/alpha	10	192	< 0.0001	+
WM 178	VGII	Clin/alpha	10	204	< 0.0001	+
WM 2540	VGI	Vet/alpha	9	228	< 0.0001	+
WM 175	VGIII	Env/alpha	9	240	< 0.0001	+
WM 2363	VGIV	Clin/alpha	5	240	< 0.0001	+
WM 2570	VGIV	Clin/alpha	5	240	< 0.0001	+
WM 02.103	VGI	Env/alpha	5	240	< 0.0001	+
WM 04.20	VGIV	Clin/alpha	4	ND	ND	+
WM 2423	VGIII	Clin/a	3	ND	ND	+
WM 05.342	VGII	Env/a	3	ND	ND	+
WM 2876	VGIV	Clin/alpha	2	ND	ND	+
CDCR272#	VGIIb	Clin/alpha	2	ND	ND	+
WM 10.17	VGIII	Vet/alpha	2	ND	ND	+
WM 2604	VGIV	Clin/alpha	1	ND	ND	+
WM 06.13	VGII	Env/alpha	1	ND	ND	+
WM 02.139	VGIII	Clin/alpha	1	ND	ND	+
WM 05.410	VGI	Clin/alpha	0	ND	ND	-
WM 08.314	VGIV	Vet/alpha	0	ND	ND	-
WM 06.634	VGII	Clin/alpha	0	ND	ND	-
WM 11.8	VGIII	Env/a	0	ND	ND	-

Clin: clinical; Env: environmental; Vet: veterinary.

*In comparison with the highly virulent strain CDCR265 (++).

# Reference strains of the highly and low virulent major sub-genotypes VGIIa (CDRC265) and VGIIb (CDRC272), respectively, of the Vancouver Island outbreak (11, 37).

NA: not applicable.

ND: no determined.

The survival curve of the larvae inoculated with the highly virulent VGIIa strain CDCR265 was used as a benchmark to determine the level of virulence of the remaining strains. Four groups of strains were thus defined and are represented with different numbers of crosses and dashes as follow: (-) strains that did not kill any larvae during the time of the experiment, (+) strains that killed at least one larva and were less virulent than the highly virulent Vancouver island outbreak VGIIa strain CDRC265 (*p* <0.05), (++) strains that were of comparable virulence as CDRC265 (*p* >0.05) and (+++) strains that were more virulent than the strain CDRC265 (*p* <0.05) (Table 2).

T-test and scatter plot graphs of *in vivo* cell and capsular sizes of each strain were generated. Laccase activity of each strain was graphed. In addition, melanin production and growth curves at 37°C were graphed per group of strains, according to their level of virulence. In all cases, *p*-values <0.05 were considered statistically significant. All statistical analysis and plots were carried out with the software Graph Pad Prism version 6 (La Jolla, CA, USA).

## Results

### Galleria mellonella virulence study

**Figure 1:**
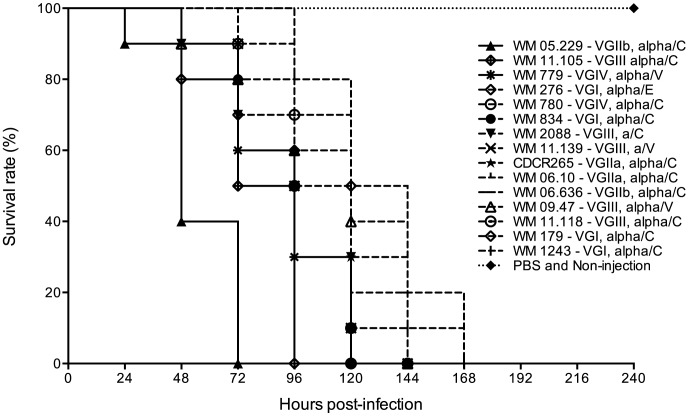
Survival curve of *Galleria mellonella* larvae inoculated with *Cryptococcus gattii*. C: clinical; E: environmental; V: veterinary. Larvae inoculated with strains more virulent (continuous line) and as virulent as the highly virulent VGIIa Vancouver Island outbreak strain CDCR265 (dashed line), as well as larvae inoculated with PBS or not inoculated (dotted line), are represented.

**Figure 2:**
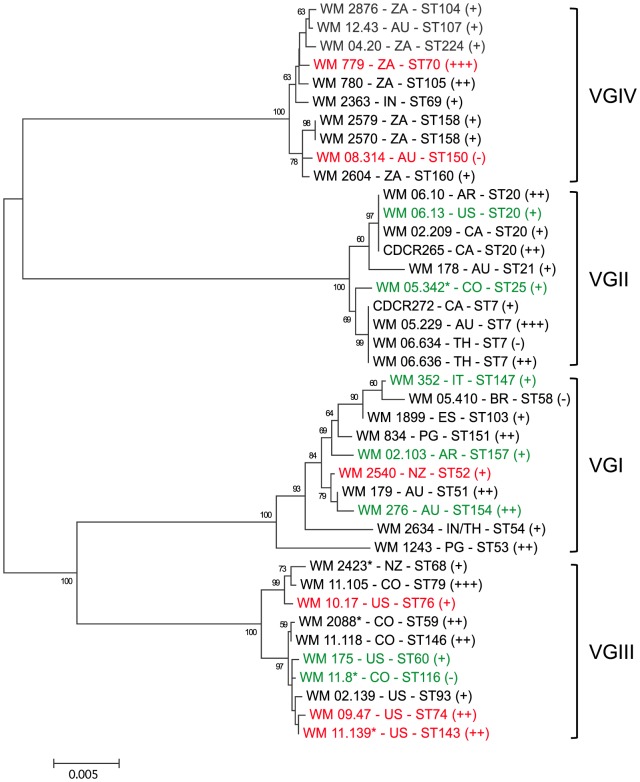
Phylogram showing the genetic relationships between the studied *Cryptococcus gattii* isolates. Dendrogram based on the maximum likelihood analysis of the concatenated seven ISHAM consensus MLST loci (8) using the program MEGA version 5.05 [51]. Bootstrap values higher than 50 are shown above the branches. Source, country, major molecular type, sequence type (ST), mating type and virulence of the strains in the *Galleria* model are indicated. Colour of the labels indicates clinical (black), veterinary (red) and environmental (green) strains; * mating type a, all others are mating type alpha; two letter country abbreviation (AR = Argentina, AU = Australia, BR = Brazil, CA = Canada, CO = Colombia, ES = Spain, IN = India, IT = Italy, NZ = New Zealand, PG = Papua New Guinea, TH = Thailand, US = USA, and ZA = South Africa); crosses represent more virulent (+++), similarly virulent (++), and less virulent strains (+), compared with the highly virulent VGIIa Vancouver Island outbreak strain CDCR265. Dash represents avirulent strains.

The inoculation of *G. mellonella* larvae with strains of all major molecular types of *C. gattii*, including the highly virulent VGIIa strain (CDCR265), resulted in rapid death of the larvae. After the first day of inoculation and incubation at 37°C, larval death was observed (Figure 1). By comparing survival curves and median survival times of the larvae inoculated with different strains, it was determined that three strains: WM 05.229 (clinical, VGII), WM 11.105 (clinical, VGIII) and WM 779 (veterinary, VGIV) were the most virulent strains, with median survival of 48, 84 and 96 h after infection, respectively. Critically, they were all significantly more virulent than the highly virulent VGIIa strain CDCR265 (median survival of 108 h (*p*<0.05)) (Figures 1 and 2, and Table 2). Eleven out of the 40 strains tested were of comparable virulence to the VGIIa strain CDCR265 (*p*>0.05), including one environmental (WM 276) and three clinical (WM 834, WM 179, WM 1243) VGI strains, two clinical (WM 06.10, WM 06.636) VGII strains; two clinical (WM 2088, WM 11.118) and two veterinary (WM 11.139, WM 09.47) VGIII strains, and one clinical (WM 780) VGIV strain. Of those, VGIII strains WM 2088 and WM 11.139 were mating type a (Table 2 and Figure 2).

Over the course of the experiment, infection of the larvae with five VGI, two VGII, one VGIII and four VGIV strains, caused at least five deaths, but all these strains were significantly less virulent than the benchmark VGIIa strain CDCR265 (*p* <0.05) (Table 2 and Figure 2).

Together with the low virulent VGIIb strain CDCR272, eight other strains killed less than five larvae, while four strains did not kill any larvae in the 10 days after infection. Therefore, they were considered to be avirulent in this model (Table 2 and Figure 2). PBS-injected larvae and uninfected controls did not die (Figure 1).

In total, 272 of the 400 inoculated larvae died. Of those, 84 larvae were killed by VGI strains, 66 by VGII, 65 by VGIII and 57 by VGIV, with median survival times of 144, 168, 144 and 240 h, respectively. Overall, strains of the molecular type VGI were responsible for a higher proportion of deaths and in less time, followed by VGIII, VGII and VGIV strains, although these differences did not reach statistical significance (*p*>0.05).

### Cellular and capsular enlargement

During *G. mellonella* larvae infection, both the cell and the capsule size of all *C. gattii* strains included in this study increased considerably, when the strains recovered from inoculated larvae were measured and compared with the same strains measured immediately before inoculation (*p* <0.001). While the total size of *C. gattii* cells before infection ranged from 5-12 µm, the total size of the cells after infection ranged from 15 µm up to 75 µm. In general, strains demonstrated a proportionally larger increase in capsule size compared to cell size (Data not shown).

**Figure 3:**
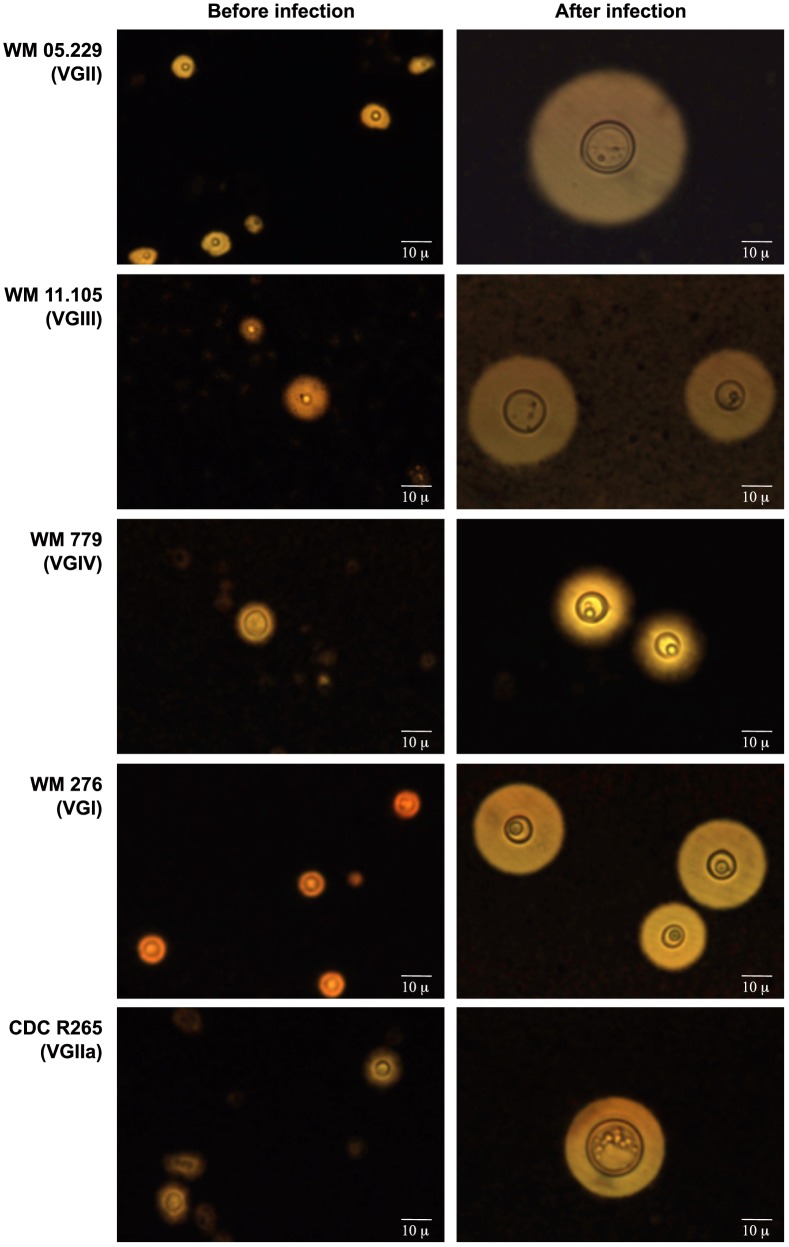
Microscopic photographs of the most virulent strain of each *Cryptococcus gattii* molecular type and CDCR265. Strains were stained with India ink before and after infection of *Galleria mellonella* larvae.

**Figure 4:**
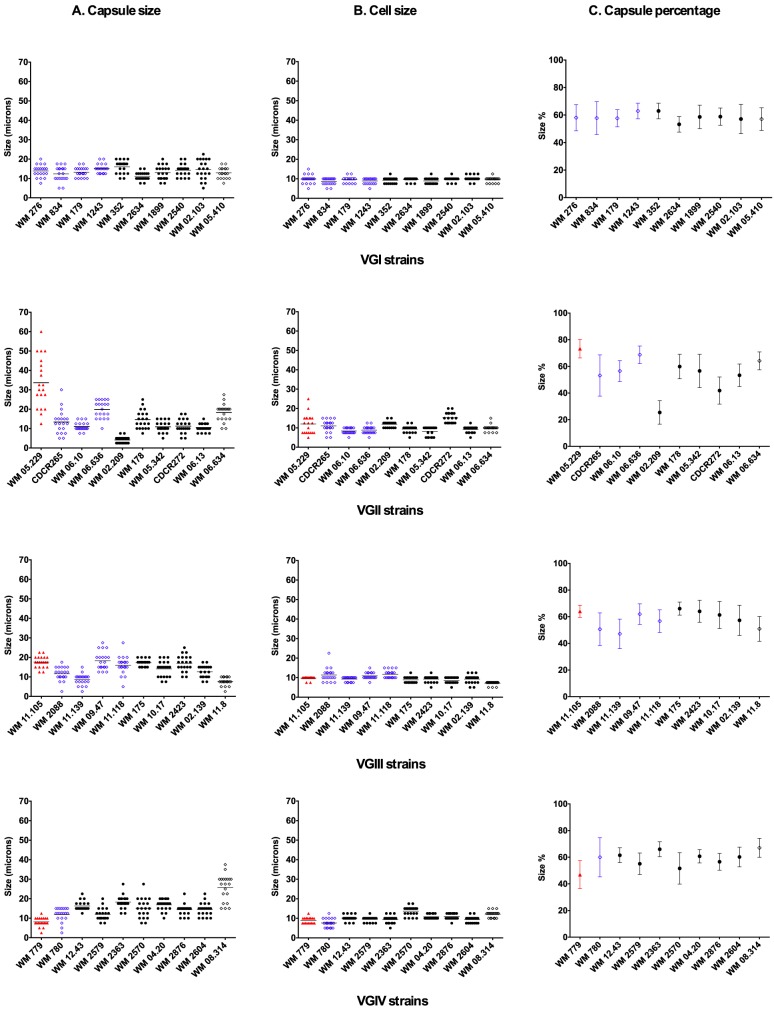
Capsule size, cell size and capsule percentage distribution of *Cryptococcus gattii* strains after larvae inoculation. Strains are grouped by major molecular type and distributed in the X-axis from more to less virulence. Symbol colour represents more virulent (red), similarly virulent (blue), and less virulent strains (black), compared with the highly virulent VGIIa Vancouver Island outbreak strain CDCR265. Empty rhombuses represent avirulent strains.

Microscopic photographs of the most virulent strain per molecular type and the highly virulent Vancouver Island outbreak VGIIa strain (CDCR265) illustrate the enormous cellular and capsular sizes reached during infection (Figure 3). Although, the most virulent strain found in this study, WM 05.229 (VGII), presented the highest average capsular size (33.6 µm) and capsule percentage (73.3%), and the fourth highest average cellular size (12.0 µm), there was no clear correlation between virulence, in terms of median survival time of the larvae, and capsular size, cellular size or capsular percentage of strains post-infection (Figure 4). Overall, VGII strains had the largest capsules followed by VGIII, VGIV and VGI strains, although this difference was not statistically significant (*p*>0.05) (Figure 4).

### Melanin production

**Figure 5:**
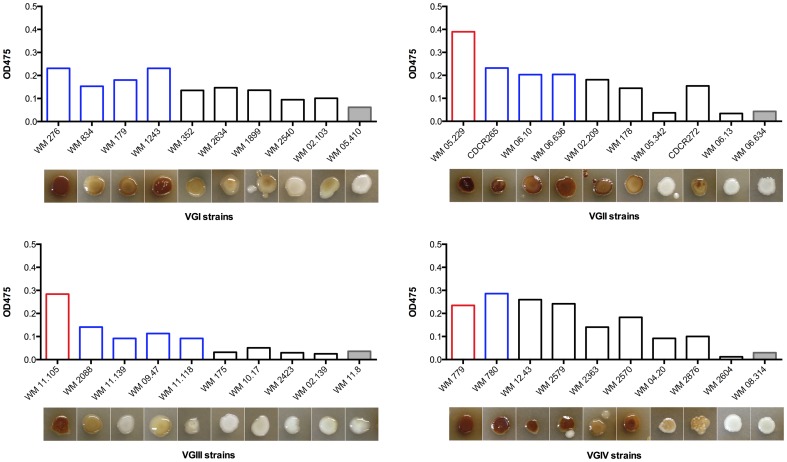
Melanin production of *Cryptococcus gattii* strains. Strains are grouped by major molecular type and distributed in the X-axis from more to less virulence. Line colour of the bars represents more virulent (red), similarly virulent (blue), and less virulent strains (black), compared with the highly virulent VGIIa Vancouver Island outbreak strain CDCR265. Grey bars represent avirulent strains. Both the quantification of the laccase activity and the ability of the strains to produce melanin in Niger seed agar are represented.

**Figure 6:**
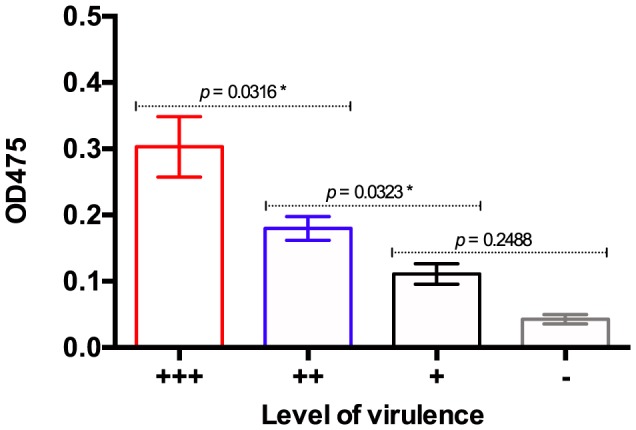
Laccase activity of *Cryptococcus gattii* strains with different level of virulence. Crosses represent more virulent (+++), similarly virulent (++), and less virulent strains (+), compared with the highly virulent VGIIa Vancouver Island outbreak strain CDCR265. Dash represents avirulent strains. More virulent strains produced more melanin than less virulent strains. *p* <0.05 was considered statistically significant.

All *C. gattii* strains in this study produced melanin (Figure 5). However, laccase activity was directly related to the virulence of the strain (*p* <0.05) (Figure 6). Melanin production was greater in the group of strains that were more virulent than CDRC265 (+++), followed by those with comparable virulence to CDCR265 (++), and by those that were less virulent than CDRC265 (+). Interestingly, strains that did not kill any larvae during the experiment (−) produced the least amount of melanin (Figure 6).

There was no correlation between the molecular type of the strains and the production of melanin (*p*>0.05).

### Growth at 37°C

**Figure 7:**
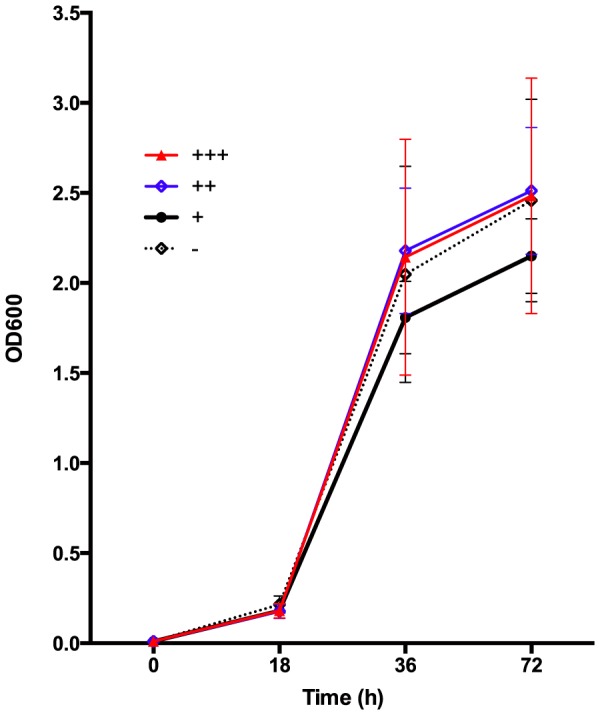
Growth rate of *Cryptococcus gattii* strains with different level of virulence at 37°C. Crosses represent more virulent (+++), similarly virulent (++), and less virulent strains (+), compared with the highly virulent VGIIa Vancouver Island outbreak strain CDCR265. Dash represents avirulent strains.

All *C. gattii* strains studied had the ability to grow at mammalian body temperature irrespective of their source of isolation. Growth rate was not correlated with either the molecular type or the level of virulence of the strains (*p*>0.05) (Figure 7).

## Discussion

Even though *C. gattii* causes a significantly less number of cases of human cryptococcosis than *C. neoformans* [2]–[4], a better understanding of the virulence of each of its major molecular types is of fundamental importance. Numerous studies, including those based on sequence analysis [9], [11], [14], mass spectra profiling [40], [41] and studies based in crosses between different molecular types (e.g. VGII and VGIII [42], support both phylogenetically and biologically the notion that each major molecular type of *C. gattii* should be recognised as an independent species.

Similarly to other studies, in which the insect model gave comparable results to those obtained with mammalian models [31]–[33], the results of this study were also consistent with those found previously using murine models to study cryptococcal virulence. *C. gattii* VGII strains that were currently found to be either more virulent (WM 05.229, WM 06.636 and WM 02.209) or less virulent (CDCR272, WM 06.13 and WM 06.634) than the highly virulent Vancouver Island outbreak strain CDCR265 in the larvae model were formerly reported as more or less virulent, respectively in a mouse model [11], [32], [37]. In the same way, the VGI strain WM 276 was shown to be highly virulent, in both insect and mouse models [11], [12], [32], [43].

As previously reported from virulence studies carried out with *C. neoformans*, the present study supports the idea that fungal virulence factors involved in pathogenesis in mammals, may also be required for infection and killing of moth larvae [44], [45]. Among those, the polysaccharide capsule which mostly contributes to the virulence of cryptococcal strains, synthesis of melanin and ability to grow at 37°C [1], [32], [46], [47]. The large capsule size produced by the strains studied in the larvae (Figure 3) has not been observed in the environment or when using capsule-inducing media during *in vitro* experimental conditions. Indeed these large capsules are reminiscent of the considerable capsule increase observed during experimental and naturally occurring pulmonary infections of mammals [44], [45], [47]. However, this phenomenon of capsule enlargement, as well as the ability to grow at 37°C was statistically similar for all strains (Figures 4 and 7), which emphasises the notion that virulence could be an emergent property of a microorganism rather than deterministic [30]. It is known that the capsule is necessary for fungal cell survival in *vivo*, but the correlation between capsule size and virulence still remains uncertain, given that strains of *C. neoformans* with very small capsules have been occasionally recovered from human and veterinary patients and acapsular strains are viable *in vitro* [47], [48]. *C. neoformans* molecular type VNI has been shown to grow more rapidly at 37°C than *C. gattii*, but no significant differences were found between the growth rate of the molecular types of *C. gattii* at this temperature [27].

Even though all studied strains synthesized melanin, this was the only virulence factor correlated with their level of virulence (Figures 5 and 6). Melanin not only contributes to the maintenance of cell wall integrity and affords protection from UV radiation, but it also enhances virulence by reducing the susceptibility of cryptococcal cells to host immune mechanisms such as phagocytosis [46], [47]. Previous studies have shown that phagocytic processes are involved in the innate immune response to fungal infection in *G. mellonella* [30], [31].

Although alpha mating-type strains have been suggested to have a higher virulence potential than mating-type a strains [1], [47], the way in which mating type affects *Cryptococcus* virulence is not completely understood. Contrary to the accepted paradigm, the current study found two highly virulent mating type a VGIII strains, one clinical (WM 2088) and one veterinary (WM 11.139). Clearly, virulence is determined by factors apart from the sexual traits of the strains. The recognized dogma that mating type alpha strains are significantly more virulent, might be biased by the considerably greater number of alpha strains recovered from both environmental and clinical samples, as alpha strains are 30- to 40-fold more predominant than the mating type a strains [4], [47].

Regardless of their source, some strains of the molecular types VGI, VGIII and VGIV, were shown to be as virulent or even more virulent than the strains of the highly virulent subtype VGIIa, responsible for several fatal cases in the ongoing Vancouver Island outbreak [10], [11] (Figures 1 and 2). Recently, three fatal cases of cryptococcosis caused by *C. gattii* molecular types VGI (two cases) and VGIII (one case), have been also reported from North America form otherwise healthy patients [12], [13], [17]. Moreover, human cases of meningitis caused by *C. gattii* molecular type VGIV, have been reported in Mexico (two patients) [22] and India (four patients) [18], as well as several cases in patients with AIDS in Sub-Saharan Africa, where the molecular type VGIV is more prevalent [20]. These finding are germane considering the association between environmental isolates and human or veterinary cases previously reported based on the identity of molecular types and genotypes found among the groups [2], [10], [11], [19], [21].

The finding that high virulent strains are present in all *C. gattii* major molecular types, is in line with the clinical observations made by others [12], [13], [17], [18], [20], [22] and supports the fact, that virulence is not specifically associated with a major molecular type, but rather related to the distinct properties of individual strains (Figure 2). The current study also revealed that two strains of the genotype VGIIb (WM 05.229 and WM 06.636) were highly virulent in the *Galleria* model, when compared with the low virulent VGIIb reference strain CDCR272 [11] (Table 2). A similar finding was reported in a previous study that compared the virulence of strains of the major *C. gattii* Vancouver Island outbreak genotypes, VGIIa and VGIIb, and demonstrated that not all strains of a specific genotype are intrinsically more or less virulent. Using a murine model, one VGIIa strain (FI623) was found to be less virulent than the highly virulent outbreak VGIIa reference strain, CDCR265 [37]. Considering that virulence depends on different structural elements and regulatory expression of various factors, as it is a complex and multi-genetic trait of pathogenic microorganisms [29], [42], [43], [46], [47], [49], strain-specific attributes that may include unique features yet not recognized, might better explain differences in virulence among the *C. gattii* strains. These findings emphasise the urgent need for combined genomic, transcriptomic and metabolomics approaches to characterise additional features of *C. gattii* that shape virulence. Such new features may represent more reliable predictors of pathogenicity in a clinical setting than the accepted measures of virulence.

In conclusion, the present study showed that virulence is not specifically associated with a particular major molecular type of *C. gattii*, but rather with individual strain attributes. In addition, it showed, that strains recovered from human and veterinary patients, the environment, and also mating type a strains can all be highly virulent. Finally, it emphasizes the value of using *G. mellonella* larvae as a substitute host, to evaluate the pathogenic potential of *C. gattii* and to cost-effectively pre-screen strains with different level of virulence. Strains of interest can then be subsequently studied in mammalian models and genotypically characterised to reveal the role that the genetic background of the strains plays in determining pathogenicity. This in turn might lead to the identification of novel virulence factors, which would represent new targets for diagnosis and treatment not only of cryptococcosis but also of other mycoses.
